# Factors associated with severe depressive symptoms among Chinese secondary school students in Hong Kong: a large cross-sectional survey

**DOI:** 10.3389/fpubh.2023.1148528

**Published:** 2023-06-06

**Authors:** Doris Y. P. Leung, Sau Fong Leung, Xue-Lin Zhang, Jia-Yin Ruan, Wing-Fai Yeung, Yim-Wah Mak

**Affiliations:** School of Nursing, The Hong Kong Polytechnic University, Kowloon, Hong Kong SAR, China

**Keywords:** academic performance, depression, secondary school, social support, substance-related disorder

## Abstract

**Background:**

Many adolescents were reported to have severe depressive symptoms, and a careful assessment of its correlates is essential for prevention and intervention programs. This study aimed to gain insight into the prevalence of severe depressive symptoms and its association with factors at four levels (individual, relationship, school and society) in a large sample of Hong Kong Chinese secondary school students.

**Methods:**

Secondary school students from Secondary 1 through 7 were selected as participants using a cluster random sampling method. A questionnaire including inventories measuring 24 factors at the four levels (six individual factors, 11 relationship factors, three school factors, and four society factors) was completed by 8,963 participants (56.3% female) with a mean age of 15.1 (SD = 1.8) years. Students with a score of ≥15 on the Patient Health Questionnaire were defined as having severe depressive symptoms. The association between severe depressive symptoms and correlates were examined by *t*-test and χ^2^ test. Logistic regression models using a hierarchical approach then examined the individual contribution of these 24 factors to severe depressive symptoms with the control of other factors in the model.

**Results:**

7.4% of the students have severe depressive symptoms. Twenty-two of the 24 factors were significantly associated with severe depressive symptoms in bivariate analyses. In the logistic regression, 11 factors (three individual factors: age, self-esteem and self-mastery; six relationship factors: tobacco use, alcohol drinking, drug use, paternal psychological control, dinner with parents, and perceived social support from friends; one school factor: felt pressure from homework; and one society factor: number of sibling) were statistically significant. Felt pressure from homework, alcohol drinking, and perceived social support from friends were the strongest correlates of severe depressive symptoms.

**Conclusion:**

The prevalence of self-reported severe depressive symptoms in Hong Kong Chinese secondary school students was high, and the identification of multiple associated factors at the four levels simultaneously provides a knowledge basis for the development of a comprehensive, multivariate model of factors influencing severe depressive symptoms in Chinese secondary school students. The factors identified in the present study may be helpful when designing and implementing preventive intervention programs.

## 1. Introduction

Adolescents face many challenges due to changes in physical growth, psychological development and future uncertainty. Some adolescents successfully cope with stressors and transit into adulthood smoothly, whereas some cannot find a way to deal with stressors and develop mental health problems including depression (1). Depression is one of the leading causes of illness and disability among adolescents (2). Onset of depression often occurs in adolescence (3, 4), and early onset depression results in an elevated risk of subsequent manifestations of psychosocial and health adversities including poor educational achievements, suicidal ideation, impaired cognitive functioning, and interpersonal problems (5–7).

The increase in adolescent depression in the last three decades is alarming. The global age-standardized prevalence of depression disorder was 89.05 per 100,000 persons in 2019, with an increase of 10.9% from 1990 (8). The global prevalence of major depressive disorder (MDD) in adolescents aged 10–19 years was 8% in a meta-analytic study (9). A critical review summarized that the estimated prevalence of MDD in adolescents in Hong Kong is low (1.3–2.2%) based on diagnostic tools (10). When non-diagnostic screening tools were used for classification, one study with 1,771 Hong Kong students (14–18 years) found an 8% prevalence of severe depressive symptoms using the Beck Depression Inventory (10), and a recent study reported that 27.4% of 9,621 adolescents had severe depressive symptoms using the Center Epidemiological Studies Depression Scale (CES-D scores ≥25) (11).

Most adolescents are studying in secondary schools, and they are different from those who are not studying in school. Students have to face unique school-related stressors, such as huge academic pressure, and burden, such as competing for academic excellence and preparing for college entrance examination. Academic pressure seems to be more influential in Chinese students, because academic excellence has been regarded as a paramount socialization goal in the Chinese society (12). Chinese parents, particularly, mothers rely more on academic performance to achieve a sense of worth, and they reported greater parental control than their Western counterparts (13); as a result, the psychological needs of their children may be neglected (12). Nonetheless, parental control can also be viewed as an expression of care and love for children in the Chinese culture, rather than an act of interference in autonomy from the Western perspective (14), making parental control a protective factor among Chinese students.

Many associated factors of depressive symptoms in adolescents have been identified in the literature. Two recent systematic reviews on Chinese adolescents concluded with a set of psychological factors relating to factors for depressive symptoms at various levels, including individual, parent, peer and school (low self-esteem, low hope, low positive affect, high rumination, poor parent–child communication, negative life events, academic pressure, abuse, poor family functioning, bullying, poor family cohesion, high parental expectation, peer support, social withdrawal and limited social network) (15, 16). Despite these research efforts, most of these prior studies had only included a few of these factors in their inquiry, which could not provide a clear picture regarding the individual contribution of these factors to depressive symptoms.

Socio-ecological Model is a theoretical model of health promotion which provides a comprehensive framework to identify factors influencing health outcomes at multiple levels (17, 18). According to the model, intra- and interpersonal factors operating within multiple ecological systems influence adolescents’ mental health. These factors include those within the individual system, relationship system, school system, community system, and society system. These social ecologies are interconnected in their relationships with mental health. Including variables related to multiple social systems in the same analysis allows for exploring how they may be inter-related.

The present study aimed to describe the rate of severe depressive symptoms and identify its associated factors based on the socio-ecological model in adolescents in a sample of Chinese secondary school students in Hong Kong. The potential effects of 24 factors in four levels of the socio-ecological model on severe depressive symptoms were examined simultaneously. These four levels include: (i) individual (6 factors: age, sex, year of study, self-esteem, self-mastery and optimism), (ii) relationships (11 factors: tobacco use, alcohol use, drug use, parent’s marital status, paternal psychological control, maternal psychological control, doing homework with parents, leisure activities with parents, dinner with parents, perceived social support from family and from friends), (iii) school (3 factors: perceived academic performance, satisfaction of academic performance and felt pressure from homework), and (v) society (4 factors: father’s educational level, mother’s educational level, number of siblings and source of family income). The findings of the study will enable the identification of adolescents who are at risk for MDD more easily, and develop interventions to improve the state of depressive symptoms.

## 2. Materials and methods

### 2.1. Study design and participants

This study is a large community-based cross-sectional study conducted among students in 16 secondary schools in Hong Kong from 2011 to 2014. In the survey (19), 16 secondary schools were randomly selected from 109 secondary schools in three districts (Tseung Kwan O, Kwun Tong and Wong Tai Sin) in Hong Kong. Students in all classes (Secondary 1 through 7) of the selected schools were invited to join the study. After consent was obtained from the school principals, the study information sheets and consent forms were delivered to parents *via* the students. Consent forms were returned if parents or students themselves refused to participate in the study. Consented students (either from themselves or their parents) were asked to complete the survey questionnaire with support from trained research assistants in a classroom setting with no teachers present. Completed and returned questionnaires were regarded as providing consent to the present study. The participants were not required to give their name in the questionnaire. A total of 12,518 students participated in the study (the refusal rate was 5.0%). Among the 12,518 completed questionnaires, 8,963 (71.4%) questionnaires with complete data in all the studied variables were included in this analysis. Among these 8,936 secondary students, their mean age was 15.1 years (SD = 1.8) and 56.3% were girls. They were even distributed in the year of study (Secondary 1: *n* = 1,186, 13.3%; Secondary 2: *n* = 1,296, 14.5%; Secondary 3: *n* = 1,555, 17.4%; Secondary 4: *n* = 1727, 19.3%; Secondary 5: *n* = 1845, 20.6%; Secondary 6: *n* = 1,223, 13.7%), except only 104 (1.2%) were in Secondary 7. Only one class in Secondary 7 was recruited in the study because of the launch of the new 6 year secondary school education system in Hong Kong in 2012. This study was approved by the Human Subjects Ethics Sub-Committee of The Hong Kong Polytechnic University and the participating organizations.

### 2.2. Measures

#### 2.2.1. Outcome variable

The outcome variable in the present study, severe depressive symptoms, was measured by the Patient Health Questionnaire (PHQ-9) (20). The nine items were rated on a 4-point Likert scale ranging from 0 (Absence) to 3 (Almost every day). The overall PHQ-9 score ranges from 0 to 27, and depressive symptoms are classified by severity into five groups, namely, minimal (scores of 0–4), mild (5–9), moderate (10–14), moderately severe (15–19), and severe (20–27). In this study, the participants were classified as having severe depressive symptoms if they had a PHQ-9 score ≥ 15, as they are likely to meet the diagnostic criteria for MDD with 95% specificity and hence may be of particular clinical concern (21). The Chinese version of PHQ-9 demonstrated good psychometric properties in adolescent samples (21, 22). The Cronbach’s alpha value of PHQ-9 in the study was 0.859.

#### 2.2.2. Individual factors

Three personal characteristics (age, sex, and year of study), and three adaptability factors (self-esteem, self-mastery and optimism) were included as individual factors in the present study. Year of study ranged from 1 (Secondary 1) to 7 (Secondary 7). Self-esteem was measured by Rosenberg Self-esteem Scale (RSE) (23). This 10-item scale was rated on a 5-point Likert scale ranging from 1 (Strongly disagree) to 5 (Strongly agree). The overall score of the scale ranges from 10 to 50, and higher scores indicate higher self-esteem level. The internal consistency of RSE in the study was 0.879. The Chinese version of the RSE is a reliable and valid measure in Chinese adolescent samples (24). The validated Chinese version of the 7-item Pearlin Mastery Scale (MAS) was used to measure individuals’ perceived self-mastery, that is, the general feeling of personal control over important life challenges (25). The scale was rated on a 5-point Likert scale from 1 (Strongly disagree) to 5 (Strongly agree), with an overall score ranging from 7 to 35. Higher overall score indicates higher level of perceived self-control. The internal consistency of the scale in the study was 0.770. The validated Chinese version of the 6-item Revised-Life Orientation Test (CLOT-R) was used to measure optimism (26). The six items were rated in a 5-point Likert scale, ranging from 0 (Strongly Disagree) to 4 (Strongly Agree). Total score ranges from 0 to 24, with higher scores indicating higher level of optimism. The Cronbach’s alpha of the scale was 0.712 in the present study.

#### 2.2.3. Relationship factors

In this study, 11 relationship-related factors were included in the analysis. Three factors are related to substance use (tobacco, alcohol, and drug use), three are family-related factors (parent’s marital status, paternal and maternal psychological control), three factors are related to nurturing parent–child relationship (doing homework, leisure activities, and dinner with parents), and two are social support factors (perceived social support from family and friends). Tobacco use was measured with one item: “Do you have a smoking habit?” The participants were classified into four groups, namely, “Never smoker,” “Current daily smoker,” “Current occasional smoker,” and “Quitter.” Alcohol use was measured with one item: “Did you drink alcohol in the past 6 months?” The participants were classified into six categories, namely, “Never drinker,” “Drink 4–6 days per week,” “Drink 1–3 days per week,” “Drink 1–3 days per month,” “Drink at least once a month on special occasions,” and “Quitter.” Drug use was measured with one item (“Have you ever used drug?”) with two response options (“Yes” or “No”). Parent’s marital status were also measured with one item with three options: “Married,” “Divorced/Separated,” and “Widowed.” The validated Chinese Paternal Psychological Control Scale (CPPCS) and Chinese Maternal Psychological Control Scale (CMPCS) measured the participants’ perceived psychological control from the father and mother, respectively (27, 28). Both scales consist of 10 items covering basic features of psychological control, including constraining verbal expression, invalidating feelings, personal attack, guilt induction, love withdrawal, and erratic emotional behaviors. All items were rated on a 4-point Likert scale from 1 (Strongly disagree) to 4 (Strongly agree). The overall score of each scale ranges from 10 to 40, with a higher score indicating a higher level of parental control. The Cronbach’s alpha values of CPPCS and CMPCS in the current study were 0.887 and 0.882, respectively. Nurturing parent–child relationship factors were measured with three separate items by asking the students to report the average time spent with parents in (i) doing homework, (ii) having leisure activities and (iii) having dinner in a week using a 5-point Likert scale ranging from 0 (Never) to 4 (Every day). Short forms of Perceived Social Support from Family Scale (PSS-Fa) and Perceived Social Support from Friends Scale (PSS-Fr) measured the participants’ social support in this study (29). PSS-Fa contains three items: “My family gives me the moral support I need,” “I rely on my family for emotional support,” and “I can talk to my family when I am sad. I would not feel bad after it.” The same items were included in PSS-Fr with the words “my family” replaced by “my friends.” Each item was scored as 0 (No) or 1 (Yes). The overall scores of PSS-Fa and PSS-Fr range from 0 to 3. A higher score indicates a higher perceived support. The Cronbach’s alpha values of PSS-Fa and PSS-Fr in this study were 0.800 and 0.795, respectively.

#### 2.2.4. School factors

Three school-related factors, namely, perceived academic performance, satisfaction with academic performance, and felt pressure from homework, were included in the analysis. All three factors were measured using one item. Perceived academic performance was assessed by asking the students to indicate their perceived poor academic performance as compared with classmates using a 5-point Likert scale ranging from 1 (Excellent) to 5 (Very poor). Satisfaction with academic performance was measured using a 4-point Likert scale ranging from 1 (Not satisfied) to 4 (Very satisfied). Pressure from homework was a dichotomous variable measured by asking the students to indicate whether they felt pressure from homework.

#### 2.2.5. Society factors

Four society factors, namely, father’s educational level, mother’s educational level, number of siblings, and source of family income, were included in the analysis. The father and mother’s educational levels were measured with 5 options from 1 (No school) to 5 (Above secondary school). Number of siblings also has 5 response options from 0 (Zero) to 4 (Four or more). Source of family income has 4 response options: “Parents,” “Relatives,” “Government aid,” and “Others.”

#### 2.2.6. Statistical analysis

Descriptive analyses were used to summarize the characteristics of the studied variables for the overall sample and for the two subgroups – those with and without severe depressive symptoms. Bivariate and multivariate analyses identified the factors associated with depressive symptoms. In the bivariate analyses, the relationships of potential associated factors with depressive symptoms were assessed by independent *t*-tests for continuous variables, *χ*^2^ tests for categorical variables. Cohen’s d for continuous variables and crude odds ratio for categorical variables were calculated to estimate the effect size. In the multivariate analysis, logistic regression was performed on the dependent variable (severe depressive symptoms) with a hierarchical block design in regression analyses to determine the separate contributions of the 24 independent variables in the four levels of factors (i.e., individual, relationship, school, and society factors) in the socio-ecological model. The first step of the hierarchical logistic regression included the six individual factors at level 1 (Model 1), the second step added the 11 relationship factors at level 2 (Model 2), the third step added the three school factors at level 3 (Model 3), and the fourth step added the four society factors at level 4 (Model 4). A hierarchical approach was employed, because it allows the investigation of the complex hierarchical relationships of different factors at different levels. In particular, distal factors could be adequately investigated without the interference of proximal factors (30). We compared the goodness of the nested models using χ^2^ difference test (Δχ^2^), and a statistically significant Δχ^2^ indicates that the addition of the regression variables could increase the goodness of fit of the model. We also reported the adjusted odds ratios (OR) and its 95% confidence intervals to estimate the strength of the association between dependent and independent variables. Data were analyzed using SPSS version 28.0 (IBM Corp., Armonk, NY, United States), and a value of *p* < 0.05 was considered statistically significant.

## 3. Results

Table 1 provides the sample characteristics in the whole sample. When compared to the score range of their respective scales, the mean score on self-esteem was on the low side while the mean scores of self-mastery and optimism were around the middle point of the scale. The majority of students (96.3%) never smoked, only 1.2% were current regular smokers, 68.0% did not drink any alcoholic drink, and 22.9% were occasional alcohol drinkers in the past 6 months. Moreover, 1.7% of the students used drugs. A total of 88.3% of the students reported that their parents were married. The students reported that the mean levels of parental and maternal psychological control were moderate, around the middle point of the scales. The mean time spent with parents in doing homework and leisure activities were on the very low side of the scale, whereas the mean time spent with parent in having dinner was on the very high side of the scale. Mean levels of perceived social support from family and friends were also on the high side of the scale. For school factors, the majority of the students (69.6%) perceived their academic performance as average or below, and 38.3% reported that they were not satisfied with their academic performance. In addition, 64% of the students reported having pressure from homework. For society factors, 49.7 and 50.2% of the students had fathers and mothers with an education level at senior secondary school or above, 23.5% were the single child in the family, and 61.25% reported that the main source of family income was from the parents. In this sample, the rates of the different severities of depressive symptoms, which were classified based on the recommendation by Kroenke et al. (20) as minimal (scores of 0–4), mild (5–9), moderate (10–14), moderately severe (15–19), and severe (20–27), were 37.7, 38.2, 16.7, 5.2, and 2.2%, respectively. The rate of having severe depressive symptoms was 7.4% in the whole sample.

**Table 1:** Sample characteristics in the whole sample.

	Total
	Total (*n* = 8,936)
**Individual factors**	
*Personal characteristics*	
Sex, *n* (%)	
Female	5,027 (56.3)
Male	3,909 (43.7)
Age, mean ± SD	15.1 ± 1.8
Year of study, *n* (%)	
Secondary 1	1,186 (13.3)
Secondary 2	1,296 (14.5)
Secondary 3	1,555 (17.4)
Secondary 4	1727 (19.3)
Secondary 5	1845 (20.6)
Secondary 6	1,223 (13.7)
Secondary 7	104 (1.2)
*Adaptability*	
Self-esteem on the RSE, mean ± SD	22.3 ± 7.0
Self-mastery on the MAS, mean ± SD	16.5 ± 4.7
Optimism on the CLOT-R, mean ± SD	12.3 ± 3.8
**Relationship factors**	
*Substance use*	
Tobacco use, *n* (%)	
Never smoker	8,604 (96.3)
Current daily smoker	105 (1.2)
Current occasional smoker	64 (0.7)
Quitter	163 (1.8)
Alcohol use in the past 6 months, *n* (%)	
Never drinker	6,075 (68.0)
Drink at least 4–6 days per week	44 (0.5)
Drink 1–3 days per week	140 (1.6)
Drink 1–3 days per month	534 (6.0)
Drink at least once a month on special occasions	2044 (22.9)
Quitter	99 (1.1)
Ever drug use, *n* (%)	
Yes	155 (1.7)
No	8,781 (98.3)
*Family*	
Parents’ marital status, *n* (%)	
Married	7,888 (88.3)
Divorced/separated	859 (9.6)
Widowed	189 (2.1)
Paternal psychological control on the CPPCS, mean ± SD	23.0 ± 5.9
Maternal psychological control on the CMPCS, mean ± SD	24.0 ± 5.9
*Nurturing parent–child relationship*	
Doing homework with parents, mean ± SD	0.54 ± 1.07
Leisure activities with parents, mean ± SD	1.6 ± 1.3
Dinner with parents, mean ± SD	3.2 ± 1.2
*Social support*	
Perceived social support-family on the PSS-Fa, mean ± SD	2.0 ± 1.2
Perceived social support-friend on the PSS-Fr, mean ± SD	2.5 ± 0.9
**School factors**	
Perceived academic performance, *n* (%)	
Excellent	261 (2.9)
Above average	2,457 (27.5)
Average	3,347 (37.5)
Below average	1832 (20.5)
Poor	1,039 (11.6)
Satisfaction of academic performance, *n* (%)	
Very satisfied	283 (3.2)
Satisfied	1,347 (15.1)
Average	3,880 (43.4)
Not satisfied	3,426 (38.3)
Felt pressure from homework, *n* (%)	
Yes	5,716 (64.0)
No	3,220 (36.0)
**Society factors**	
*Family resources*	
Father’s education level, *n* (%)	
No school	134 (1.5)
Primary school	1,527 (17.1)
Junior secondary school	2,836 (31.7)
Senior secondary school	3,522 (39.4)
Above secondary school	917 (10.3)
Mother’s education level, *n* (%)	
No school	211 (2.4)
Primary school	1,518 (17.0)
Junior secondary school	2,718 (30.4)
Senior secondary school	3,755 (42.0)
Above secondary school	734 (8.2)
Number of siblings, *n* (%)	
0	2097 (23.5)
1	4,571 (51.2)
2	1,497 (16.8)
3	449 (5.0)
4+	222 (2.5)
Source of family income, *n* (%)	
Parents	8,370 (61.2)
Relatives	63 (27.3)
Government aid	368 (7.1)
Others	389 (4.4)
**Depressive symptoms**	
Minimal	3,368 (37.7)
Mild	3,418 (38.2)
Moderate	1,493 (16.7)
Severe	364 (5.2)
Very severe	194 (2.2)

RSE, Rosenberg Self-Esteem Scale; MAS, Pearlin Mastery Scale; CLOT-R, Chinese version of Revised Life Orientation Test; CPPCS, Chinese Paternal Psychological Control Scale; CMPCS, Chinese Maternal Psychological Control; PSS-Fa, Perceived Social Support from Family Scale; PSS-Fr, Perceived Social Support from Friends Scale.

Table 2 shows the bivariate associations of the studied factors with severe depressive symptoms. The results of bivariate analyses showed that 22 out of the 24 included factors were associated with severe depressive symptoms in the secondary school student sample. The two exemptions were sex and mother’s educational level. Overall, the students with severe depressive symptoms were those who were older in age; in more senior year of study; had lower levels in self-esteem, self-mastery, and optimism; had tobacco, illicit drug, and alcohol use; had divorced/separated parents; had higher levels of parental psychological control; had spent less time in doing homework, leisure activities, and dinner with parents, had lower levels of social support from family and friends; had perceived poor level in academic performance, lower level of satisfaction in academic performance, had pressure from homework; had fathers with lower educational level; had more siblings; and had source of family income other than parents. The data of the hierarchical logistic regression for severe depressive symptoms are presented in Table 3. The final model (Model 4) was considered as the best competing model, as indicated by the statistically significance of the Δχ^2^ test statistics. Model 4 shows that 11 out of the 24 studied factors were statistically significant. Seven factors, namely, age, tobacco use, alcohol use in the past 6 months, drug use, paternal psychological control, felt pressure from homework, and number of siblings were positively and significantly associated with severe depressive symptoms, whereas four factors, namely, self-esteem, self-mastery, dinner with parents, and perceived social support from friends were negatively and significantly associated with severe depressive symptoms. The result indicated that students who were older (*p* = 0.003); current daily smoker (*p* = 0.021); had alcohol drinks 1–3 days per week (*p* < 0.001) or 1–3 days per month (*p* < 0.001) in the past 6 months; had drug use (*p* = 0.031); had more paternal psychological control (*p* = 0.004); felt pressure from homework (*p* < 0.001); had more siblings (*p* = 0.003); had lower levels of self-esteem (*p* < 0.001), self-mastery (*p* < 0.001), and perceived social support from friends (*p* < 0.001); and had lesser time spent in dinner with parents (*p* < 0.001) reported larger odds of having severe depressive symptoms. Among the 11 significant factors, alcohol use on a regularly basis and pressure from homework were the two strongest predictive factors, whereas perceived social support from friends was the strongest protective factor for severe depressive symptoms. All the ORs of the independent variables remained stable across the four models except the year of study, which became insignificant after the addition of the other factors in the model.

**Table 2:** Bivariate associations of severe depressive symptoms with factors in the socio-ecological model.

	Severe depressive symptoms				
	Yes (*n* = 657)	No (*n* = 8,279)	χ^2^/*t* statistics	value of *p*	Odds ratio/Cohen’s d
**Individual factors**					
*Personal characteristics*					
Sex, n (%)			0.529^a^	0.467	
Female	379 (57.7)	4,648 (56.1)			0.939^c^
Male	278 (42.3)	3,631 (43.9)			Reference
Age, mean ± SD	15.45 ± 1.81	15.10 ± 1.79	4.885^b^	<0.001	0.198^d^
Year of study, mean ± SD	3.86 ± 1.65	3.64 ± 1.64	3.317^b^	<0.001	0.134^d^
*Adaptability*					
Self-esteem on the RSE, mean ± SD	13.71 ± 6.30	22.97 ± 6.62	−34.644^b^	<0.0001	−1.404^d^
Self-mastery on the MAS, mean ± SD	10.64 ± 4.35	16.95 ± 4.38	−35.517^b^	<0.0001	−1.440^d^
Optimism on the CLOT-R, mean ± SD	8.62 ± 3.92	12.58 ± 3.61	−26.934^b^	<0.001	−1.092^d^
**Relationship factors**					
*Substance use*					
Tobacco use, *n* (%)			107.405^a^	<0.001	
Never smoker	591 (90.0)	8,013 (96.8)			Reference
Current daily smoker	30 (4.6)	75 (0.9)			5.423^c^
Current occasional smoker	16 (2.4)	48 (0.6)			4.519^c^
Quitter	20 (3.0)	143 (1.7)			1.179^c^
Alcohol use in the past 6 months, *n* (%)			132.907^a^	<0.001	
Never drinker	355 (54.0)	5,720 (69.1)			Reference
Drink at least 4–6 days per week	10 (1.5)	34 (0.4)			4.739^c^
Drink 1–3 days per week	33 (5.0)	107 (1.3)			4.969^c^
Drink 1–3 days per month	77 (11.7)	457 (5.5)			2.715^c^
Drink at least once a month on special occasions	174 (26.5)	1870 (22.6)			1.499^c^
Quitter	8 (1.2)	91 (1.1)			1.416^c^
Ever drug use, *n* (%)			11.884^a^	< 0.001	Reference
Yes	23 (3.5)	132 (1.6)			2.239^c^
No	634 (96.5)	8,147 (98.4)			
*Family*					
Parents’ marital status, *n* (%)			9.241^a^	0.010	
Married	556 (84.6)	7,332 (88.6)			Reference
Divorced/Separated	84 (12.8)	775 (9.4)			1.429^c^
Widowed	17 (2.6)	172 (2.1)			1.303^c^
Paternal psychological control on the CPPCS, mean ± SD	25.75 ± 7.29	22.77 ± 5.74	10.244^b^	<0.001	0.508^d^
Maternal psychological control on the CMPCS, mean ± SD	26.66 ± 6.88	23.78 ± 5.78	10.435^b^	<0.001	0.490^d^
*Nurturing parent–child relationship*					
Doing homework with parents, mean ± SD	0.31 ± 0.86	0.56 ± 1.08	−6.862^b^	<0.001	−0.229^d^
Leisure activities with parents, mean ± SD	1.10 ± 1.19	1.61 ± 1.31	−10.510^b^	<0.001	−0.391^d^
Dinner with parents, mean ± SD	2.71 ± 1.36	3.21 ± 1.15	−9.150^b^	<0.001	−0.429^d^
*Social support*					
Perceived social support-family on the PSS-Fa, Mean ± SD	1.23 ± 1.26	2.08 ± 1.14	−16.833^b^	<0.001	−0.744^d^
Perceived social support-friend on the PSS-Fr, mean ± SD	1.89 ± 1.27	2.57 ± 0.87	−18.569^b^	<0.001	−0.753^d^
**School factors**					
Perceived academic performance, mean ± SD	2.30 ± 1.18	2.94 ± 1.00	−13.450^b^	<0.001	−0.631^d^
Satisfaction of academic performance, mean ± SD	1.42 ± 0.74	1.86 ± 0.79	−14.875^b^	<0.001	−0.568^d^
Felt pressure from homework, *n* (%)			136.216^a^	<0.001	
Yes	559 (85.1)	5,157 (62.3)			3.453^c^
No	98 (14.9)	3,122 (37.7)			Reference
**Society factors**					
*Family resources*					
Father’s education level, mean ± SD	3.33 ± 0.99	3.40 ± 0.93	−1.958^b^	0.038	−0.084^d^
Mother’s education level, mean ± SD	3.32 ± 1.03	3.37 ± 0.93	−1.326^b^	0.185	−0.059^d^
Number of siblings, mean ± SD	1.25 ± 1.01	1.10 ± 0.89	3.851^b^	<0.001	0.173^d^
Source of family income, *n* (%)			27.973^a^	<0.001	
Parents	584 (88.9)	7,786 (94.0)			Reference
Relatives	9 (1.4)	54 (0.7)			2.222^c^
Government aid	45 (6.8)	323 (3.9)			1.857^c^
Others	19 (2.9)	116 (1.4)			2.184^c^

a χ^2^ test.

b Independent *t*-test.

c Odds ratio.

d Cohen’s d.

RSE, Rosenberg Self-Esteem Scale; MAS, Pearlin Mastery Scale; CLOT-R, Chinese version of Revised Life Orientation Test; CPPCS, Chinese Paternal Psychological Control Scale; CMPCS, Chinese Maternal Psychological Control; PSS-Fa, Perceived Social Support from Family Scale; PSS-Fr, Perceived Social Support from Friends Scale.

**Table 3:** Outcome of hierarchical regression analysis of severe depressive symptoms in the sample of Hong Kong Chinese secondary students (*n* = 8,936).

	Model 1 Adjusted OR (95% CI)	Model 2 Adjusted OR (95% CI)	Model 3 Adjusted OR (95% CI)	Model 4 Adjusted OR (95% CI)
**Individual factors**				
*Personal characteristics*				
Male (female as reference)	0.991 (0.823–1.194)	0.865 (0.712–1.050)	0.904 (0.744–1.099)	0.929 (0.763–1.130)
Age	1.278 (1.145–1.425)***	1.202 (1.072–1.348)**	1.216 (1.083–1.364)**	1.193 (1.055–1.342)**
Year of study	0.832 (0.738–0.938)**	0.894 (0.788–1.015)	0.870 (0.765–0.988)*	0.879 (0.773–1.000)
*Adaptability*				
Self-esteem on the RSE	0.884 (0.866–0.903)***	0.889 (0.871–0.908)***	0.893 (0.874–0.913)***	0.893 (0.873–0.913)***
Self-mastery on the MAS	0.813 (0.790–0.838)***	0.831 (0.807–0.856)***	0.835 (0.810–0.860)***	0.835 (0.810–0.860)***
Optimism on the CLOT-R	0.973 (0.942–1.006)	0.985 (0.952–1.019)	0.987 (0.955–1.021)	0.986 (0.953–1.020)
**Relationship factors**				
*Substance use*				
Tobacco use (never smoker as reference)				
Current daily smoker		2.126 (1.177–3.840)*	2.204 (1.218–3.990)**	2.040 (1.116–3.728)*
Current occasional smoker		1.420 (0.689–2.925)	1.478 (0.712–3.065)	1.491 (0.712–3.120)
Quitter		0.996 (0.544–1.821)	1.063 (0.579–1.951)	1.025 (0.558–1.882)
Alcohol use in the past 6 months (never drinker as reference)				
Drink as least 4–6 days per week		0.427 (0.139–1.749)	0.471 (0.152–1.461)	0.491 (0.154–1.561)
Drink 1–3 days per week		2.526 (1.522–4.192)**	2.606 (1.569–4.329)***	2.669 (1.604–4.442)***
Drink 1–3 days per month		1.751 (1.266–2.421)***	1.785 (1.289–2.471)***	1.826 (1.316–2.532)***
Drink at least once a month on special occasions		1.142 (0.912–1.430)	1.146 (0.915–1.436)	1.164 (0.928–1.460)
Quitter		0.761 (0.331–1.749)	0.774 (0.335–1.785)	0.803 (0.346–1.865)
Ever drug use		2.038 (1.098–3.780)*	2.067 (1.113–3.841)*	1.995 (1.066–3.732)*
*Family*				
Parent’s marital status (married as reference)				
Divorced/separated		0.980 (0.731–1.314)	0.976 (0.727–1.311)	0.915 (0.674–1.242)
Widowed		1.312 (0.723–2.379)	1.403 (0.773–2.544)	1.161 (0.616–2.185)
Paternal psychological control on the CPPCS		1.028 (1.010–1.047)**	1.027 (1.009–1.046)**	1.027 (1.009–1.045)*
Maternal psychological control on the CMPCS		1.009 (0.990–1.027)	1.007 (0.988–1.026)	1.007 (0.989–1.026)
*Nurturing parent–child relationship*				
Doing homework with parents		0.929 (0.824–1.047)	0.928 (0.823–1.047)	0.924 (0.819–1.043)
Leisure activities with parents		0.988 (0.900–1.084)	0.990 (0.902–1.087)	0.986 (0.898–1.082)
Dinner with parents		0.862 (0.798–0.931)***	0.862 (0.798–0.932)***	0.862 (0.797–0.932)***
*Social support*				
Perceived social support-family on the PSS-Fa		0.928 (0.852–1.011)	0.928 (0.715–1.010)	0.937 (0.860–1.021)
Perceived social support-friend on the PSS-Fr		0.785 (0.721–0.854)***	0.778 (0.715–0.848)***	0.781 (0.717–0.850)***
**School factors**				
Perceived academic performance			1.034 (0.933–1.146)	1.035 (0.933–1.147)
Satisfaction of academic performance			0.890 (0.754–1.050)	0.875 (0.742–1.033)
Felt pressure from homework			1.614 (1.253–2.081)***	1.519 (1.255–2.089)***
**Society factors**				
*Family resources*				
Father’s educational level				1.007 (0.898–1.128)
Mother’s educational level				1.088 (0.969–1.222)
Number of sibling				1.166 (1.054–1.289)**
Source of family income (parent as reference)				
Relatives				1.376 (0.897–2.109)
Government aid				1.501 (0.642–3.514)
Others				1.419 (0.783–2.572)
Nagelkerke *R*^2^	0.349	0.382	0.386	0.390
Model Δχ^2^ (Δdf)	1374.544 (6)	143.272 (18)	17.289 (3)	14.621 (6)
value of p for model Δχ^2^	< 0.001	< 0.001	< 0.001	0.023

*p < 0.05; **p < 0.01; ***p < 0.001. CI, confidence interval; OR, odds ratio; RSE, Rosenberg Self-Esteem Scale; MAS, Pearlin Mastery Scale; CLOT-R, Chinese version of Revised Life Orientation Test; CPPCS, Chinese Paternal Psychological Control Scale; CMPCS, Chinese Maternal Psychological Control; PSS-Fa, Perceived Social Support from Family Scale; PSS-Fr, Perceived Social Support from Friends Scale.

## 4. Discussion

In the present study, we found that the rate of severe depressive symptoms was 7.4%, which was comparable to the global estimate of clinical depression (8%) in adolescents (9). In Hong Kong, a previous review on local studies observed that the prevalence of depressive symptoms in adolescents varied substantially across studies using screening tools, in which PHQ-9 tended to report a lower prevalence compared with CES-D (10). PHQ-9 was designed to diagnose and assess the severity of MDD based on a diagnostic tool for depression, the fourth edition of the Diagnostic and Statistical Manual of Mental Disorders, thus the low observed rate of severe depressive symptoms based on PHQ-9 in the current study is reasonable. Nevertheless, the high prevalence of adolescent depressive symptoms in Hong Kong secondary school students is alarming and deserves attention (11, 16).

The present study differed from most previous studies among Chinese adolescents in that the association of severe depressive symptoms with contributing factors was examined from a board list of factors at multiple levels. The findings of the present study indicate that pressure from homework and alcohol drinking are the strongest contributing factors to severe depressive symptoms. In line with findings from a systematic review of Chinese adolescents in mainland China (15), pressure from homework was the most prominent factor contributing to severe depressive symptoms among secondary students in Hong Kong in the multivariate analysis. A previous local study published in 1999 also reported that the amount of schoolwork is associated strongly with depressive symptoms in Grade 9 and 10 students in Hong Kong (31). In the Chinese culture, effort is valued as a primary factor in educational success; thus, much emphasis has been placed on spending more time in doing a large amount of homework in achieving better academic results compared with students in other countries (32–34). Consistent with previous literature (15, 31, 35), we also found that poor academic performance and low satisfaction were significantly associated with severe depressive symptoms in the bivariate analysis but they became statistically non-significant in the hierarchical logistic regression. The result suggested their influences on severe depressive symptoms might have been shared by other factors in the socio-ecological model. For interventions to alleviate depressive symptoms from pressure of homework as well as to motivate students to learn better, teachers could take an active role to raise students’ activity and content interests by assigning high-quality homework and providing an atmosphere to motivate students in growing their responsibility for their school learning (36).

We found that the three types of substance use are associated with severe depressive symptoms but to different extents, with alcohol drinking as the strongest associated factor. The findings were consistent with previous literature had reported both cigarette smoking and alcohol drinking are significantly associated with depressive symptoms and the relationships were bi-directional in longitudinal studies (37–40). The weak associations observed in smoking and drug use as compared to that in alcohol drinking could be due to the legal restriction on substance use in Hong Kong. For smoking, selling tobacco products to any persons under 18 was prohibited in 1994 and smoking was completely banned in almost indoor public places and in all schools in 2007; but for alcohol, a reduced tax for most types of alcoholic beverages was imposed to promote economic activities in 2007 (41, 42). The low observed prevalence of tobacco and drug use, might have led to the weak associations in tobacco use and drug use in this study. Nevertheless, our study was the first include all three types of substance use as correlates of severe depressive symptoms in secondary school students for examination in Hong Kong, and produced valuable information that students who had a habit of any substance use are at risk of having severe depressive symptoms and need assistance.

Social support from friends was correlated negatively with severe depressive symptoms but social support from family was not correlated with severe depressive symptoms in our multivariate analysis. Previous literature evidenced the importance of support from friends and peer by providing protection from social–emotional distress but the increasing salience of peers and friends can also impact negatively on the nature and quality of non-peer relationships, especially family relationship (43, 44). The relative dependence is transferred from parents to friends during this transition period as adolescents start to form their own peer circle and tend to disengage from parental control for more autonomy. The non-significance of social support from family in the current study however was contradictory to the meta-analytic findings among secondary school in China, which reported that support from friends and family are of equal importance in secondary students in mainland China (15). This discrepancy could be explained, at least partially, by the inclusion of other parent-related variables in the current analysis. In Tang et al.’s review (15), the effect size of the factors was calculated based on zero-order correlation coefficient without adjustment for any covariates. In our analysis, we included eight parent-related factors. Among them, two factors, paternal psychological control and time spent in having dinner with parents, remained statistically significant in the multivariate analysis. The significant parent-related variables possibly overlapped with social support from family and explained the shared variations in the dependent variable severe depressive symptoms in adolescents. The significant findings in these two parent-related variables highlighted their importance in students’ depressive symptom development in Hong Kong. A 3-year longitudinal study reported that parents in Hong Kong devoted more attention to the academic study of their children than other aspects of development although the psychological control from parents declined over time (45). As academic excellent is the paramount socialization goal, overemphasis on academic excellence would create stress and conflict for the parents, the children, and the family; and would finally lead to depressive symptoms in the children. Thus, it has been advocated to launch programs targeting parents to help them accept the academic limitations of their children and cope in a healthy manner are advocated (12).

An important finding from the current study is that students who spend more time in dinner with parents were less likely to have severe depressive symptoms. Hong Kong is famous for its busy life that parents in Hong Kong spend very little time with their children. Fathers in Hong Kong only spend an average of 6 min a day with their children in an early study in 2007 (46) and the situation should be even worse recently given that both parents had to work and at a longer time to earn a living. Perhaps, the students considered having dinner with parents as an expression of care of love from their parents in such a busy city. This finding suggests that spending time to do some ordinary things together could be a good way to alleviate severe depressive symptoms in Hong Kong secondary students.

We also found that self-esteem and self-mastery were independently associated with severe depressive symptoms in the multivariate analysis. Previous studies consistently reported the buffering effects of positive cognitive development (16, 47–49). Our study findings further confirmed the importance of positive cognitive development on severe depressive symptoms using a large sample covering secondary students from early to late adolescents in Hong Kong. In addition, age, and number of siblings were related to severe depressive symptoms in the present study. The findings suggested that older students and students with more siblings were more vulnerable to severe depressive symptoms. Previous literature suggested that age 13 is a turning point for depression, and girls reported higher depression levels than boys after age 13 (47). As we have recruited a large secondary student sample with most of the students aged over 13, we observed the significant results in age. Regarding the number of siblings, previous studies in China had focused on the prevalence rates of depressive symptoms in adolescents in single-child families versus those in non-single-child families, and mixed results were reported (47, 50–52). Our findings echoed some of the studies reporting that the rates of depressive symptoms were higher in non-single-child families (52, 53). The mixed results in these previous studies might be due to the dichotomy of the variable, the number of siblings, which reduced its variability in the sample. Our study differed from those in previous studies as our participants had a wide range in the number of siblings, from zero sibling to more than four siblings, which provided variations in this variable for examining its association with depressive symptoms. Further investigation could focus on the moderating effects of these demographic characteristics on the associations of severe depressive symptoms with factors at the individual, relationship, and school levels.

Our study has some limitations. First, the use of a cross-sectional design limits the extent to which causal inferences of the observed associations can be established. The findings should be further examined in longitudinal studies to establish the temporal validity of any associations found. In addition, given the cross-sectional nature of the study, the results referred to the state aspect of severe depressive symptoms but not the trait aspect. Second, it was entirely based on self-report data. Students may under-report their depressive symptoms because of social desirability. In addition, the use of PHQ-9 as a screening tool for measuring severe depressive symptoms may be another limitation as nothing can be said about the prevalence of clinical depression in our sample. Third, the scales measuring perceived social support from family and from friends (PSS-Fa and PSS-Fr) have not been validated in Chinese samples before its adoption in the current study. This factor might have introduced measurement bias in the study findings, although the two scales showed high reliability in the study. Fourth, some of the factors were measured with one item, which might have introduced measurement bias in the study findings. Further studies should consider validated tools in measuring the studied variables. Moreover, we did not collect the information of some important factors of severe depressive symptoms in the study. For example, no information was obtained about specific life stressors which may paly confounding roles in the examination.

## 5. Conclusion

In summary, this large-scale study found that the prevalence of severe depressive symptoms was high in Hong Kong secondary school students, and 11 multiple factors at the individual, relationships, school, and society levels were associated with severe depressive symptoms in secondary school students. The findings provides a knowledge basis from which to develop strategic depression prevention programs among Hong Kong Chinese secondary school students. Early monitoring of the possibility of severe depressive symptom should target secondary school students with adverse family characteristics such as those with high psychological control from father, busy parents, or many siblings. Individual or group intervention should be implemented for secondary school students with regular substance use, low self-esteem or self-mastery, had pressure from homework, or low perceived social support from friends.

## Data availability statement

The datasets presented in this article are not readily available because permission to use the dataset by other researchers has to seek approval from the ethical committee. Requests to access the datasets should be directed to yw.mak@polyu.edu.hk.

## Ethics statement

The studies involving human participants were reviewed and approved by Departmental Research Committee, School of Nursing, The Hong Kong Polytechnic University. Written informed consent to participate in this study was provided by the participants’ legal guardian/next of kin.

## Author contributions

YW-M designed and implemented the study. DL carried out the analysis, interpreted the results and drafted the manuscript. SL, X-LZ, J-YR, W-FY, and Y-WM assisted in drafting and reviewing the manuscript. All authors contributed to the article and approved the submitted version.

## Funding

This study was funded by Central Research Grant, The Hong Kong Polytechnic University (G-YL13).

## Conflict of interest

The authors declare that the research was conducted in the absence of any commercial or financial relationships that could be construed as a potential conflict of interest.

## Publisher’s note

All claims expressed in this article are solely those of the authors and do not necessarily represent those of their affiliated organizations, or those of the publisher, the editors and the reviewers. Any product that may be evaluated in this article, or claim that may be made by its manufacturer, is not guaranteed or endorsed by the publisher.
